# Transition to Robotic Total Knee Arthroplasty With Kinematic Alignment is Associated With a Short Learning Curve and Similar Acute-Period Functional Recoveries

**DOI:** 10.7759/cureus.38872

**Published:** 2023-05-11

**Authors:** Zachary S Morrisey, Matthew F Barra, Paul G Guirguis, Christopher J Drinkwater

**Affiliations:** 1 Orthopedic Surgery, University of Rochester School of Medicine and Dentistry, Rochester, USA; 2 Orthopedic Surgery, University of Rochester Medical Center, Rochester, USA; 3 Orthopedics, University of Rochester School of Medicine and Dentistry, Rochester, USA

**Keywords:** computer navigation, learning curve, acute recovery, knee arthroplasty, surgical robotics

## Abstract

Background

Robotic instruments are increasingly being used in total knee arthroplasty (TKA). The adoption of robotics has allowed surgeons a new level of precision and facilitated the adoption of a kinematic approach in TKA. We sought to examine one surgeon’s transition from a traditional mechanical alignment technique to a modified kinematic approach by comparing short-term recovery outcomes of robotic TKA patients with those who underwent traditionally instrumented TKAs.

Methodology

We examined six-week and six-month postoperative data from 99 traditionally instrumented, mechanically aligned and 66 kinematically aligned robotic TKA patients between January 2021-October 2021 and October 2021-April 2022, respectively. Robotic surgery was performed with VELYS™ (DePuy Synthes, Warsaw, IN, USA) a semi-active, imageless, table-affixed, robotic TKA solution.

Results

Robotic and traditionally instrumented TKAs did not differ significantly in any functional outcome measures examined, including pain scores, use of assistive devices, or range of motion at six weeks postoperatively. Robotic TKA patients had a better range of motion in knee flexion than traditional TKA patients at six months postoperatively. There were no differences in surgical complications or rates of manipulation under anesthesia within one year postoperatively. Robotic surgery tourniquet times exhibited a steep drop off and equaled traditional methods after only two robotic surgeries were performed.

Conclusions

Transition to a kinematic, semi-active, robotic TKA demonstrated encouraging results by demonstrating acute-period recovery of function consistent with the current standard of care, as well as a better range of motion at six months postoperatively. The learning curve of this new-to-market device was shorter than previous research on the transition to robotic TKA. Clear advantages of transitioning to robotic instrumentation by any specific functional measure are yet to be elucidated. Further randomized trials are necessary to characterize long-term outcomes.

## Introduction

Robotic surgical devices have displayed promising results in various surgical fields due to their ability to operate more precisely than even the best-trained surgeons. Total knee arthroplasty (TKA) robots can follow surgical plans that are determined by examining patient anatomy preoperatively with radiographs (image-based systems) or intraoperatively by target mapping bony landmarks (imageless systems). During TKA, robotic devices prevent tibial or femoral cuts that deviate from the surgical plan; thus, these devices facilitate more precise joint alignment angles that are in line with the planned joint axis [1]. Clinical trials have shown robotic instrumentation to increase the function of the joint and patient satisfaction postoperatively [2]. In addition to improvements in patient satisfaction, reviews have also found imageless computer navigation, the basis for imageless robotics, to substantially reduce the rate of revision surgery [3]. Many robotic solutions have not been available long enough for long-term prosthesis survival to be determined. Additionally, robotic-assisted TKA has been found to improve soft tissue balance, achieve better flexion-extension gaps, and reduce iatrogenic injury to periarticular soft tissue envelope compared to conventional TKA [1,4]. Robotic TKA remains a heated debate; some randomized controlled trials have not found robotics to improve long-term outcomes [5], and the available robotic TKA solutions have many differences among them.

The implementation of robotics may be limited initially by the training and learning curve, which manifests itself in extended operative times. An analysis of surgeons transitioning to robotic approaches showed that surgeons took 6-11 surgeries to learn the new techniques and stabilize surgery times [6]. Additionally, a study evaluating the learning curve of a popular image-based robotic solution found a learning curve of 16 surgeries across three different mediums for high-volume surgeons [7]. Previous research has shown that among high-volume arthroplasty surgeons, robotics significantly increase a surgeon’s time in the operating room [8]. However, this research is mixed, with some reviews showing that surgeons achieve operative times similar to conventional jig-based methods after around 15 robotic operations [9]. Overall, robotic TKA has shown some promise in short-term outcomes and in reducing outliers by improving surgical precision but has also been shown to be associated with significant learning curves and an overall extension of operation times.

Our study objective was to determine the effect that transitioning to a novel robotic surgical approach to TKA had on short-term patient recoveries. Additionally, we sought to examine the learning curve of this new-to-market robotic solution and determine differences if any from established research on the transition to robotics. To our knowledge, this study is the first to examine an organic clinical transition to this specific device. Our hypothesis has the following two main elements: transitioning to robotic instrumentation with kinematic alignment would improve short-term patient recoveries, and the device learning curve would be similar to other robotic solutions.

## Materials and methods

Study design

This was a retrospective review of medical records examining patients who received either robot-guided and kinematically aligned TKA procedures or traditionally instrumented mechanically aligned TKA procedures. This study received a patient informed consent exemption from the University of Rochester Office for Human Subject Protection on May 31, 2022 (Study ID: STUDY00007211). These surgeries and the subsequent data collection occurred from January 2021 to October 2021 for the traditionally instrumented TKA procedures and from October 2021 to April 2022 for the robotic instrumentation TKA procedures. This time frame represents the study surgeon’s transition from traditional to robotic TKA, with the complete transition for most TKA cases occurring in early November 2021. All TKAs were performed by the senior author and used the same implant design, the Depuy Attune Cruciate Retaining implant (DePuy Synthes, Warsaw, IN, USA). Robotic instrumentation was conducted with the VELYS™ robotic solution (DePuy Synthes, Warsaw, IN, USA). This robotic solution is new to the market, having just been FDA 501k approved for use in early 2021.

Inclusion criteria included patients receiving primary TKA by either traditional instrumentation with mechanical alignment or robotic instrumentation with kinematic alignment. Exclusion criteria included revision surgery, age below 30 or above 89, lack of six-week follow-up data in the form of patient office or telemedicine visit note, prior major knee trauma or arthritis related to traumatic injury, unexpected major deformity to the knee, and unavailability of patient medical records.

We identified 69 robotic primary TKAs and 105 traditionally instrumented TKA patients within the study duration. Three robotic and three traditional TKA patients had prior previous major trauma to the knee, and three traditional TKA patients did not have a follow-up at six weeks postoperatively. All identified primary TKA patients were between the ages of 30 and 89, leaving a final sample size of 66 robotic TKA patients and 99 traditionally instrumented TKA patients. We examined typical clinical indicators of short-term recovery following TKA, namely, goniometer-measured range of motion in degrees of flexion and extension, patient use of assistive devices for mobility, pain level, and other patient-reported outcome measures, such as depression scores. All of these data points were collected as standard practice at the patients’ six-week follow-up visit. We also examined rates of surgical complications, including the incidence of infection and hospital readmission within the first six weeks postoperatively. Patient knee pain score was determined by patient self-report of their pain on a 1-10 visual analog scale at their six-week postoperative follow-up. Finally, we examined operative tourniquet time as a proxy measure for surgical efficiency.

Surgical techniques

The traditional technique was measured resection, addressing the femur first. Femoral instrumentation was anterior referencing using an intramedullary guide set at 5° valgus for varus knees and 3-4° valgus for valgus knees. Femoral component rotation referenced the line parallel to the transepicondylar axis. Tibial preparation utilized an extramedullary referencing guide set at neutral, with a 5-7° slope, determined by preoperative radiographic assessment. A standard median parapatellar approach was employed. Balancing and final polyethylene component selection were performed during trial placement.

Robotic-assisted, modified kinematic technique aimed to preserve final mechanical alignment between 3° varus and valgus, per long-established recommendations regarding mechanical alignment following TKA [10]. A median parapatellar approach was also used, but all anticipated balancing was performed before any cuts to create a mostly symmetric initial balance curve within 2 mm of neutrality. Starting values for coronal and sagittal plane tibial cuts and coronal plane femoral cuts were modified to replicate the original anatomy based on calculations from preoperative long-leg standing and lateral knee X-rays. Rotation was calculated by balancing the medial and lateral flexion spaces, once the surgical approach and initial soft tissue releases were complete and accepting the same posterolateral gap or 0.5 mm larger than the posteromedial gap. Final bone cuts were then adjusted on the robot’s operative planning screen to create balance curve neutrality in flexion and extension while maintaining a planned mechanical axis within the aforementioned parameters. Because the TKA device is an imageless robotic solution, bony landmarks were mapped and tracked intraoperatively with positional sensors that communicate with a tower that tracks the sensor’s position in a three-dimensional space.

Statistical analysis

All data collection and statistical analyses were performed using Microsoft Excel® software. Data normality for all continuous variables was established with the Shapiro-Wilk test, with an alpha value of less than 0.05 signifying non-normality. Reported p-values for normally distributed continuous variables are two-tailed t-tests assuming equal variances. Reported p-values for non-normally distributed continuous variables are Mann-Whitney U tests. P-values reported for categorical variables are chi-squared test values. A p-value of 0.05 was used as the cutoff for statistical significance. Power analysis revealed that a sample size of at least 126 subjects would be required to detect a 5° difference in ROM, assuming a two-sided alpha of 0.05 and a desired power of 0.8, and a sample of 80 patients would be required to detect a five-minute change in average operation time. Based on this assessment, we examined patients in the aforementioned time parameter, with a final sample of 165 patients.

## Results

There were no differences in relevant patient demographics/biometrics between groups (Table 1).

**Table 1:** Patient demographics. Age and body mass index are reported as mean ± SD. Sex is reported as N female (% female).

Characteristic	Robotic (N = 66)	Traditional instrumentation (N = 99)	P-value
Age	68.21 ± 6.91	69.52 ± 7.17	0.25
Sex	35 (53.0)	56 (56.6)	0.65
Body mass index	30.27 ± 4.62	30.88 ± 5.28	0.45

A summary of the comparison between traditional instrumentation and robotic instrumentation is presented in Table 2. Indicators of early functional postoperative recovery showed no significant differences between groups at six weeks postoperatively. Pain levels (p = .08), range of motion (p = .28), and patient use of assistive devices at six weeks postoperatively (p = 0.15) were not different. Importantly, there were no differences in postoperative infections or differences in the rate of emergency department/office visits or hospital readmissions within six weeks postoperatively (p = 0.68). Tourniquet times, as a proxy measure for the length of surgery and surgical efficiency, were not different between groups (p = 0.34). Interestingly, traditionally instrumented TKA patients had greater decreases in their PROMIS depression scores postoperatively compared to their preoperative levels (p = 0.02). Pre to postoperative PROMIS function score differences were not significant (p = 0.31). Further data from patient follow-up (Table 3) revealed that robotic TKA patients had better knee flexion at six months postoperatively (p = 0.01). Rates of manipulation under anesthesia within one year of operation were not different between traditional and robotic TKA patients (p = 0.88).

**Table 2:** Early functional recovery of robotic total knee arthroplasty versus traditional instrumentation. Use of assistive devices reported as N using assistive devices at six wks (% using assistive devices). Emergency department or hospital visits reported as N who had emergency department visits or admissions (% of emergency department visits or admissions). All others are reported as mean ± SD.

Variable	VELYS™ (N = 66)	Traditional instrumentation (N = 99)	P-value
Range of motion flexion at six weeks (degrees)	110.15 ± 11.3	107.83 ± 13.30	0.28
Range of motion extension at six weeks (degrees)	1.87 ± 2.97	2.69 ± 3.68	0.35
Pain level at six weeks	1.80 ± 2.12	2.20 ± 1.86	0.08
Use of assistive devices	26 (40)	49 (51.6)	0.15
Tourniquet time (minutes)	64.66 ± 9.15	63.46 ± 10.62	0.34
Emergency department or hospital admission within six weeks	13 (20)	17 (17.2)	0.68
PROMIS Depression Difference (score)	-.81 ± 5.33	3.13 ± 7.14	0.02
PROMIS Function Difference (score)	-.33 ± 5.33	-1.76 ± 5.47	0.31

**Table 3:** Comparison of six-month range of motion and one-year rate of manipulation under anesthesia between robotic and traditional total knee arthroplasty. Manipulation under anesthesia (MUA) reported as N requiring MUA within one year postoperatively (%). Range of motion reported as mean ± SD.

Variable	VELYS™ (N = 66)	Traditional instrumentation (N = 99)	P-value
Range of motion flexion at six months (degrees)	117.41 ± 8.06	114.04 ± 11.20	0.01
Manipulation under anesthesia within one year	3 (4.5)	5 (5.1)	0.88

Data comparing the early to late transition to the robot are summarized in Table 4. An examination of acute-period recoveries from the early robotic transition compared to the late robotic transition period yielded virtually identical results. The first 20 surgeries completed with the robot showed no short-term recovery differences with the following 46 surgeries in knee pain (p = 0.83), range of flexion (p = 0.53), or use of assistive devices (p = 0.09). The first 20 robotic surgeries had no difference in tourniquet times compared to the following 46 surgeries (p = 0.42).

**Table 4:** Early versus late robotic transition. Use of assistive devices reported as N using assistive devices at six weeks (% using assistive devices). Emergency department or hospital visits reported as N who had emergency department visits or admissions (% of emergency department visits or admissions). All others are reported as mean ± SD.

Variable	VELYS™ early transition (N = 20)	VELYS™ Late Transition (N = 46)	P-value
Range of motion flexion (degrees)	110.25 ± 24.45	110.10 ± 11.98	0.53
Range of motion extension (degrees)	1.43 ± 1.77	2.07 ± 3.28	0.88
Pain level	1.75 ± 1.77	1.82 ± 2.27	0.83
Use of assistive devices	11 (55)	15 (32.6)	0.09
Tourniquet time (minutes)	66.00 ± 17.21	64.01 ± 8.16	0.42
Emergency department or hospital admission within six weeks	3 (15)	10 (21)	0.53

Surgery times with the robotic system were similar to traditional instrumentation after the first two surgeries performed. Tourniquet times from robotic surgeries three through 10 were no different from surgeries 11 through 66 (Table 5).

**Table 5:** Analysis of tourniquet times. Reported as mean ± SD (p-value compared to the reference).

Variable	First two robot operations (N = 2)	Robot operations 3–10 (N = 8)	Robot operations 11–66 (N = 56)	Traditional instrumentation (N = 99)
Tourniquet time (minutes)	91.0 ± 7.07 (p = 0.0004)	65.63 ± 5.53 (p = 0.57)	63.48 ± 8.11 (p = 0.99)	63.46 ± 10.62 (ref)

**Figure 1:**
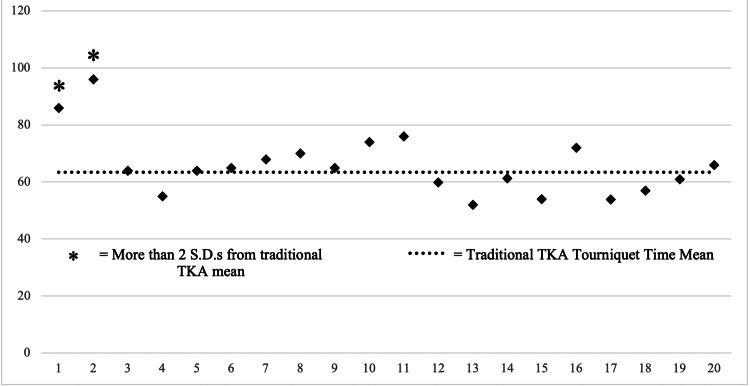
First 20 robotic TKA tourniquet times vs. traditional TKA tourniquet time mean Time in minutes. TKA: total knee arthroplasty

Surgery times exhibited a steep drop following the second surgery and essentially equalized with traditional instrumentation methods thereafter (Figure 1).

## Discussion

The robot under investigation in this study is unique in that it offers the ability to use a kinematic alignment technique or the traditional mechanical alignment. The study surgeon chose to use kinematic alignment. The current standard in TKA is to restore alignment to a neutral mechanical axis or to a marginally valgus axis. However, in patients presenting with constitutional varus alignment, restoring their joint to a neutral mechanical axis may deviate their limb alignment unnaturally [11]. Constitutionally, varus knees and other non-neutral alignments make up roughly 80% of the general population, with just 20% having truly neutral anatomical alignment [12]. Kinematic alignment aims to maintain the constitutional alignment of the patient to prevent the discomfort or unintended mechanical consequences of altering a patient’s anatomical alignment. A study regarding the complication rates following TKA determined that kinematic alignment did not increase the rate of complication rate postoperatively compared to conventional TKA [13]; additionally, kinematic alignment has demonstrated higher Knee Society Scores than conventional methodology [13,14], and improved functional outcomes including increased flexion in the long term [15]. Some long-term trials, however, have found no difference in patient outcome measures between kinematic and mechanical alignment [16]. The myriad of robotic TKA solutions available complicates the kinematic versus mechanical debate, as both the definition of a truly kinematic alignment and surgical techniques varies from study to study; traditional jig-based manual instrumentation is much more standardized by comparison.

Our data suggest two key findings. First, a robotically instrumented, kinematically aligned approach to TKA demonstrated short-term recovery as well as manually instrumented, mechanically aligned TKAs. Second, imageless, semi-active robotic instrumentation demonstrated a short learning curve for our study surgeon.

Robotic instrumentation with kinematic alignment had similar short-term recovery as traditional methods. The ability of a patient to walk unassisted is one of the key features of proper recovery following TKA. As such, discontinuation of assistive devices is a litmus test to gauge patient pain and recovery of function. The two patient groups used assistive devices at similar rates six weeks postoperatively, indicating that robotic instrumentation with kinematic alignment did not significantly change patients’ short-term functional status. Additionally, recovery of the full range of motion is another key short-term recovery indicator that was not different between robotic and traditional methods, indicating a similar regain of function. Typical recovery goals for TKA propose that patients should be able to achieve 115° of knee flexion after knee replacement. Previous research has shown that achieving a range of motion of 105° of flexion by one-month post-TKA is associated with meeting the long-term range of motion goals [17]. Conversely, it has been shown that failing to meet these short-term flexion goals is associated with poor joint flexion at one-year postoperatively [17]. Our data suggest that, on average, both traditionally instrumented, mechanically aligned and robotically instrumented, kinematically aligned TKA patients met the accepted short-term recovery goals. Moreover, the finding that patients’ self-reported pain was not different further solidified that acute-period recoveries were similar. Range of motion was demonstrated to be better in the robotic TKA group at six months postoperatively. However, the clinical significance of this difference is uncertain. Finally, the finding that both groups had no differences in the rate of manipulation under anesthesia within one year postoperatively further solidified the similarity in outcomes. In sum, it did not appear that the theoretical improvements in precision from robotic instrumentation or theoretical improvements from kinematic alignment had detectable effects on acute-period functional recoveries at six weeks, and conferred small range of motion improvements at six months.

Previous research has established that surgeons require around 15 uses with robotic instrumentation to achieve similar surgery times to their well-practiced traditional methods [9]. Additionally, a recent analysis of high-volume arthroplasty surgeons has found that the transition to robotic instrumentation substantially increased surgery times, even over the long term [8]. Studies on the transition to other robotic TKA solutions found learning curves of anywhere between six and 16 surgeries [6,7]. In our study, robotic surgery times were equal to traditional instrumentation after just two robotic surgeries (Figure 1 and Table 5); unlike the studies previously mentioned, we did not see a gradual reduction in robotic surgery times with an eventual plateau. The reduction in surgery times had a well-demarcated cutoff after two surgeries and was not well described by either a linear or exponential reduction pattern. This represents a significant improvement from established research, suggesting a device learning curve that is shorter than other devices and improved surgical efficiency. These findings are of the utmost clinical importance. Surgeons’ fears that robotic technology will significantly impact their workflow remains one of the top barriers to the adoption of new technology. Additionally, recovery data from the first 20 *critical transition* robotic TKA patients were no different than the next 46, indicating that any learning curve from the new device had no impact on patients’ acute surgical recoveries. Equal outcomes were achieved starting from the first surgeries. This addresses the common concern that learning a new technique would impact patient recovery, one that dissuades surgeons of all types from adopting new techniques.

The first and most salient limitation of this study was its retrospective nature. Patients were not randomly assigned to groups. Hence, while we found no differences in major patient biometrics/demographics, it cannot be said with certainty that there were no differences between patients receiving traditional or robotic TKAs. The retrospective nature of this study was a benefit in some ways. Primarily because the analysis of the surgeon’s transition was organic and represents how a surgeon’s transition to a new technique would normally occur in clinical practice. The study design of only using one surgeon’s transition also confers strengths and limitations. It limits generalizability to the greater population of arthroplasty surgeons, as this surgeon’s specific skillset and aptitude for robotic methods may be different than other surgeons. However, this design did greatly increase the internal validity of our findings. Examining a single surgeon’s data reduces variability, guaranteeing uniformity of surgical techniques. Additionally, the study design examined only acute-period functional recovery and did not have available data for preoperative values for the range of motion, joint alignment, or pain. We cannot make any claims regarding the effects of these surgical techniques or devices on implant longevity or long-term outcomes. Additionally, we cannot make claims regarding causality. Finally, statistical power may have been limited due to the sample size. While continuous variables were well-powered for this study, categorical variables require much larger samples for adequate power. This was limited by this study’s goal of examining a specific smaller transitional period of patients.

## Conclusions

Acute-period functional recoveries following robotically instrumented and kinematically aligned TKAs were similar to those from traditionally instrumented TKAs. Patients receiving kinematically aligned, robotic TKAs had better knee flexion at six months postoperatively, although the clinical significance of this difference is yet to be established. Imageless, semi-active robotic instrumentation demonstrated a much shorter learning curve in a single-surgeon analysis than previous research on robotic transitions has shown. This study, while retrospective, remains highly clinically relevant. Analysis of new-to-market surgical technologies to determine any differences in patient functional outcomes, complication rates, and operative efficiency is critical information for other surgeons considering the implementation of these devices and techniques into their practice.
